# Crystal structure of a tetrameric RNA G-quadruplex formed by hexanucleotide repeat expansions of C9orf72 in ALS/FTD

**DOI:** 10.1093/nar/gkae473

**Published:** 2024-06-11

**Authors:** Yanyan Geng, Changdong Liu, Naining Xu, Monica Ching Suen, Haitao Miao, Yuanyuan Xie, Bingchang Zhang, Xueqin Chen, Yuanjian Song, Zhanxiang Wang, Qixu Cai, Guang Zhu

**Affiliations:** Clinical Research Institute of the First Affiliated Hospital of Xiamen University, Fujian Key Laboratory of Brain Tumors Diagnosis and Precision Treatment, Xiamen Key Laboratory of Brain Center, the First Affiliated Hospital of Xiamen University, School of Medicine, Xiamen University, Xiamen, Fujian, China; Institute for Advanced Study and State Key Laboratory of Molecular Neuroscience, Division of Life Science, The Hong Kong University of Science and Technology, Clear Water Bay, Kowloon, Hong Kong SAR, China; Department of Neurosurgery and Department of Neuroscience, Fujian Key Laboratory of Brain Tumors Diagnosis and Precision Treatment, Xiamen Key Laboratory of Brain Center, the First Affiliated Hospital of Xiamen University, School of Medicine, Xiamen University, Xiamen, Fujian, China; Institute for Advanced Study and State Key Laboratory of Molecular Neuroscience, Division of Life Science, The Hong Kong University of Science and Technology, Clear Water Bay, Kowloon, Hong Kong SAR, China; HKUST Shenzhen Research Institute, Hi-Tech Park, Nanshan, Shenzhen, Guangdong, China; Institute for Advanced Study and State Key Laboratory of Molecular Neuroscience, Division of Life Science, The Hong Kong University of Science and Technology, Clear Water Bay, Kowloon, Hong Kong SAR, China; HKUST Shenzhen Research Institute, Hi-Tech Park, Nanshan, Shenzhen, Guangdong, China; Institute for Advanced Study and State Key Laboratory of Molecular Neuroscience, Division of Life Science, The Hong Kong University of Science and Technology, Clear Water Bay, Kowloon, Hong Kong SAR, China; HKUST Shenzhen Research Institute, Hi-Tech Park, Nanshan, Shenzhen, Guangdong, China; Institute for Advanced Study and State Key Laboratory of Molecular Neuroscience, Division of Life Science, The Hong Kong University of Science and Technology, Clear Water Bay, Kowloon, Hong Kong SAR, China; Department of Neurosurgery and Department of Neuroscience, Fujian Key Laboratory of Brain Tumors Diagnosis and Precision Treatment, Xiamen Key Laboratory of Brain Center, the First Affiliated Hospital of Xiamen University, School of Medicine, Xiamen University, Xiamen, Fujian, China; Department of Neurosurgery and Department of Neuroscience, Fujian Key Laboratory of Brain Tumors Diagnosis and Precision Treatment, Xiamen Key Laboratory of Brain Center, the First Affiliated Hospital of Xiamen University, School of Medicine, Xiamen University, Xiamen, Fujian, China; Clinical Research Institute of the First Affiliated Hospital of Xiamen University, Fujian Key Laboratory of Brain Tumors Diagnosis and Precision Treatment, Xiamen Key Laboratory of Brain Center, the First Affiliated Hospital of Xiamen University, School of Medicine, Xiamen University, Xiamen, Fujian, China; Jiangsu Key Laboratory of Brain Disease Bioinformation, Department of Genetics, Xuzhou Medical University, Xuzhou, Jiangsu, China; Department of Neurosurgery and Department of Neuroscience, Fujian Key Laboratory of Brain Tumors Diagnosis and Precision Treatment, Xiamen Key Laboratory of Brain Center, the First Affiliated Hospital of Xiamen University, School of Medicine, Xiamen University, Xiamen, Fujian, China; State Key Laboratory of Vaccines for Infectious Diseases, School of Public Health, Xiamen University, Xiamen, Fujian, China; Institute for Advanced Study and State Key Laboratory of Molecular Neuroscience, Division of Life Science, The Hong Kong University of Science and Technology, Clear Water Bay, Kowloon, Hong Kong SAR, China; HKUST Shenzhen Research Institute, Hi-Tech Park, Nanshan, Shenzhen, Guangdong, China

## Abstract

The abnormal GGGGCC hexanucleotide repeat expansions (HREs) in *C9orf72* cause the fatal neurodegenerative diseases including amyotrophic lateral sclerosis and frontotemporal dementia. The transcribed RNA HREs, short for r(G4C2)_n_, can form toxic RNA foci which sequestrate RNA binding proteins and impair RNA processing, ultimately leading to neurodegeneration. Here, we determined the crystal structure of r(G4C2)_2_, which folds into a parallel tetrameric G-quadruplex composed of two four-layer dimeric G-quadruplex via 5′-to-5′ stacking in coordination with a K^+^ ion. Notably, the two C bases locate at 3′- end stack on the outer G-tetrad with the assistance of two additional K^+^ ions. The high-resolution structure reported here lays a foundation in understanding the mechanism of neurological toxicity of RNA HREs. Furthermore, the atomic details provide a structural basis for the development of potential therapeutic agents against the fatal neurodegenerative diseases ALS/FTD.

## Graphical Abstract

**Graphical Abstract:**
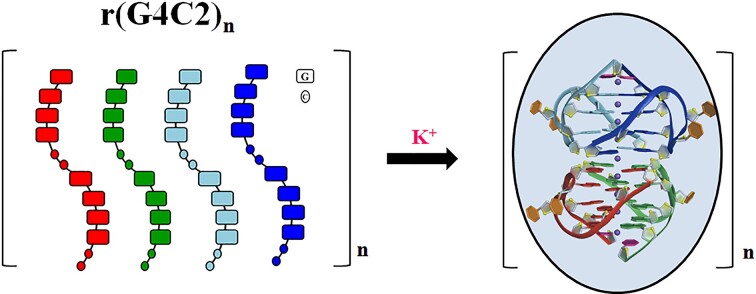

## Introduction

Amyotrophic lateral sclerosis (ALS) and frontotemporal dementia (FTD) are two distinct yet related neurodegenerative disorders that share overlapping clinical and pathological features (1,2). ALS is a progressive and fatal disorder characterized by the selective degeneration of lower and upper motor neurons, resulting in motor function impairment (3). Whereas FTD refers to a heterogeneous group of neurodegenerative disorders characterized by the progressive degeneration of the frontal and/or temporal lobes of the brain (4). It has been identified that the *C9orf72* gene mutation, characterized by the hexanucleotide repeat expansions (HREs) of GGGGCC (G4C2)_*n*_ sequence located in the first non-coding region of *C9orf72* gene, is the most common genetic cause of familial ALS and FTD (5–7). Interestingly, this mutation leads to the formation of various complex secondary structures such as G-quadruplexes (8–11). These structures have been implicated in the pathogenesis of ALS/FTD through a distinct mechanism associated with their structure polymorphism (12,13).

G-quadruplexes are secondary structures formed by the stacking of two or more G-tetrads in guanine-rich regions of DNA or RNA (14). Each G-tetrad consists of four guanine bases linked by Hoogsteen hydrogen bonds and stabilized by monovalent cations (such as K^+^ and Na^+^) (15,16). G-quadruplexes can exist as intramolecular structures formed by a single-stranded DNA or RNA molecule, or as intermolecular structures involving multiple independent nucleic acid strands. They exhibit polymorphic and can adopt parallel, antiparallel, or hybrid topologies (17). Accumulated evidences indicate that the aberrant expansion of short nucleotide repeats can cause many neurodegenerative diseases such as (CTG)_*n*_/(CAG)_*n*_ in Huntington disease (HD) and Spinocerebellar Ataxia (SCA), (CGG)_*n*_ in Fragile X syndrome (FXS) and (G4C2)_*n*_ in ALS/FTD (18,19). In particular, these nucleotide repeats can fold into specific secondary structures such as hairpin form adopted by (CTG)_*n*_/(CAG)_*n*_ (20) and G-quadruplex formed by (CGG)_*n*_ and (G4C2)_*n*_ (21). Therefore, the understanding of structural mechanism is critical in finding the treatments of fatal diseases caused by these nucleotide repeats.

Significant progress has been made in understanding the mechanisms underlying ALS/FTD, leading to the proposal of three main mechanisms: the accumulation of toxic RNAs with repeat sequences (22,23), loss-of-function of the *C9orf72* encoded proteins (24), and the production of toxic peptides and proteins through a process called non-ATG translation (25,26). r(G4C2)_*n*_ has been observed to be able to form RNA gels via phase separation (27) and the specific G-quadruplex structure was found to trigger its phase transition (28), emphasizing the important role of G-quadruplex in RNA-driven phase separation. Additionally, the non-ATG translation of r(G4C2)_*n*_ leads to the production of toxic polypeptides that are toxic to neurons (11,29,30). It has been reported that RNA helicase DHX36 selectively binds to and unwinds the RNA G-quadruplex in r(G4C2)_*n*_, facilitating the non-ATG translation process (31). This finding suggests that targeting the G-quadruplex structure could be a potential strategy for regulating the production of toxic peptides. Furthermore, the presence and structure of RNA have been shown to influence the aggregation of peptides and proteins through phase separation (32,33). Therefore, obtaining high-resolution structural information about the r(G4C2)_*n*_ G-quadruplex is crucial for understanding the pathogenesis of ALS/FTD and developing more effective therapeutic approaches (34,35).

Here, we reported the crystal structure of an eight-layer parallel tetrameric RNA G-quadruplex formed by two repeats of *C9orf72* HRE RNA, r(G4C2)_2_, in K^+^ solution. The structure of r(G4C2)_2_ is composed of two parallel propeller-type G-quadruplexes, which are formed by two r(G4C2)_2_ molecules. Intriguingly, two dimeric unit stacks into a tetramer via 5′-to-5′ mode, in which the G1 base in one dimeric unit stacks with the G7 base in the opposite dimeric unit. The observed 5′-to-5′ stacking mode is same as one of the stacking mode of d(G4C2)_2_ G-quadruplex that we reported previously (36). Interestingly, in addition to the two CC double-chain-reversal loops, the two cytosine bases located at the 3′ end of each stand stacks with the outer G-tetrad layer via π–π stacking in coordination of two K^+^ ions. The crystal structure reported here not only expands the structural polymorphism of HREs in *C9orf72*, but also provide an excellent model for drug discovery targeting ALS/FTD

## Materials and methods

### Sample preparation

The single RNA strands were obtained from Integrated DNA Technologies (IDT) and dissolved in buffer containing 70 mM KCl and 20 mM potassium phosphate (pH 7.0) with a concentration of 0.1 mM for the single strand. The strands were then annealed by heating to 95°C for 15 min, followed by slow cooling to room temperature overnight. For the samples used for crystallization, the RNA samples were further purified by FPLC using a mono Q column (Cytiva). The binding buffer is 70 mM KCl and 20 mM potassium phosphate (pH 7.0) and the eluting buffer is 1M KCl and 20 mM potassium phosphate (pH 7.0). The samples were loaded to the mono Q column and eluted by the high salt. With the increasing concentration of KCl, different conformations of RNA samples were separated into several fractions. The fraction of main peak were concentrated and exchanged into a buffer containing 20 mM Tris, 100 mM KCl (pH 7.0) after characterized by NMR method (36,37).

### NMR spectroscopy

Nuclear magnetic resonance (NMR) experiments were performed on 500 MHz and 800 MHz Varian spectrometers at 25°C. The concentration of RNA sample was typically around 0.1 mM.

### Circular dichroism spectroscopy

Circular dichroism (CD) spectra were recorded using an JASCO J-810 CD spectrometer at 25°C with a 1 mm path length quartz cuvette and a sample volume of 400 μl. The RNA oligonucleotides were prepared at concentration of 15 μM for the single strand.

### CD melting

The CD melting experiments were performed with a temperature range from 25°C to 95°C at 0.2°C/min. The concentration of RNA oligonucleotides was at 50 μM for the single strand. The CD signal were measured at a single wavelength and then normalized by using the equation (CD signal − min)/(max − min), in which CD signal is the absorbance at a given temperature, max is the maximum absorbance at 260 nm (for parallel G-quadruplexes) and min is the minimum value. Data was fit by the Boltzmann sigmoid equation (GraphPad Prism).

### Polyacrylamide gel electrophoresis (PAGE)

Non-denaturing PAGE was conducted using a 20% polyacrylamide gel (acrylamide:bis-acrylamide 29:1), supplemented with 20 mM KCl in both the gel and running buffer (0.5× TBE). The samples were prepared at a single strand concentration of 0.1 mM. Gels were stained with a red-safe dye.

### Size exclusion chromatography coupled with multi-angle light scattering (SEC-MALS)

The SEC-MALS system consists of an HPLC system (Agilent), a static light scattering detector (Wyatt), and a differential refractive index detector (Agilent). 100 μl sample with the concentration of 200 μM was loaded by autosampler (Agilent) into a Superose 12 10/300 column pre-equilibrated with the buffer containing 70 mM KCl and 20 mM potassium phosphate (pH 7.0). Data were analyzed by ASTRA7 (Wyatt).

### Crystallization

The r(G4C2)_2_ sample at concentration of 1.5 mM was initially screened using the Nucleic Acid Mini Screen kit (Hampton Research) with the hanging-drop vapour-diffusion technique at 16°C. Small crystals were grown from a reagent containing 40 mM Sodium Cacodylate trihydrate (pH 7.0), 10% MPD, 12 mM spermine tetrahydrochloride and 80 mM KCl with reservoir of 50% MPD. The condition was further optimized by varying the concentration of samples and MPD, as well as the temperature for crystallization. Ultimately, high-quality crystals suitable for diffraction were obtained from reagent containing 40 mM Sodium Cacodylate trihydrate (pH 7.0), 10% MPD, 12 mM spermine tetrahydrochloride, 80 mM KCl with the reservoir containing 35% MPD at a sample concentration of 2 mM at 4°C within 1 day.

### Data collection and structure determination

The crystals of r(G4C2)_2_ were flash-cooled in liquid nitrogen for data collection. The diffraction data sets were collected on beamlines BL19U at the Shanghai Synchrotron Radiation Facility (SSRF). Diffraction data were processed using the HKL2000 packages (38). The structure was solved by molecular replacement using one chain from the crystal structure of d(G4C2)_2_ (PDB: 7ECH) as the searching model by Phaser (39). Manual model building and refinement were performed iteratively using COOT (40) and Refmac5 (41). The final refinement statistics are summarized in Table 1. All figures of G-quadruplex structure were prepared using PyMOL (http://www.pymol.org).

**Table 1.** The *T*_m_ values of r(G4C2G4), r(G4C2)_2_ and r(G4C2)_4_ in 35 mM and 70 mM KCl solution

	r(G4C2G4)	r(G4C2)_2_	r(G4C2)_2_
35 mM KCl	∼84.0°C	∼78.1°C	∼81.6°C
70 mM KCl	∼82.2°C	∼81.1°C	∼81.8°C

## Results

### Parallel G-quadruplex formed by r(G4C2)_*n*_ in K^+^ solution

**Figure 1.**
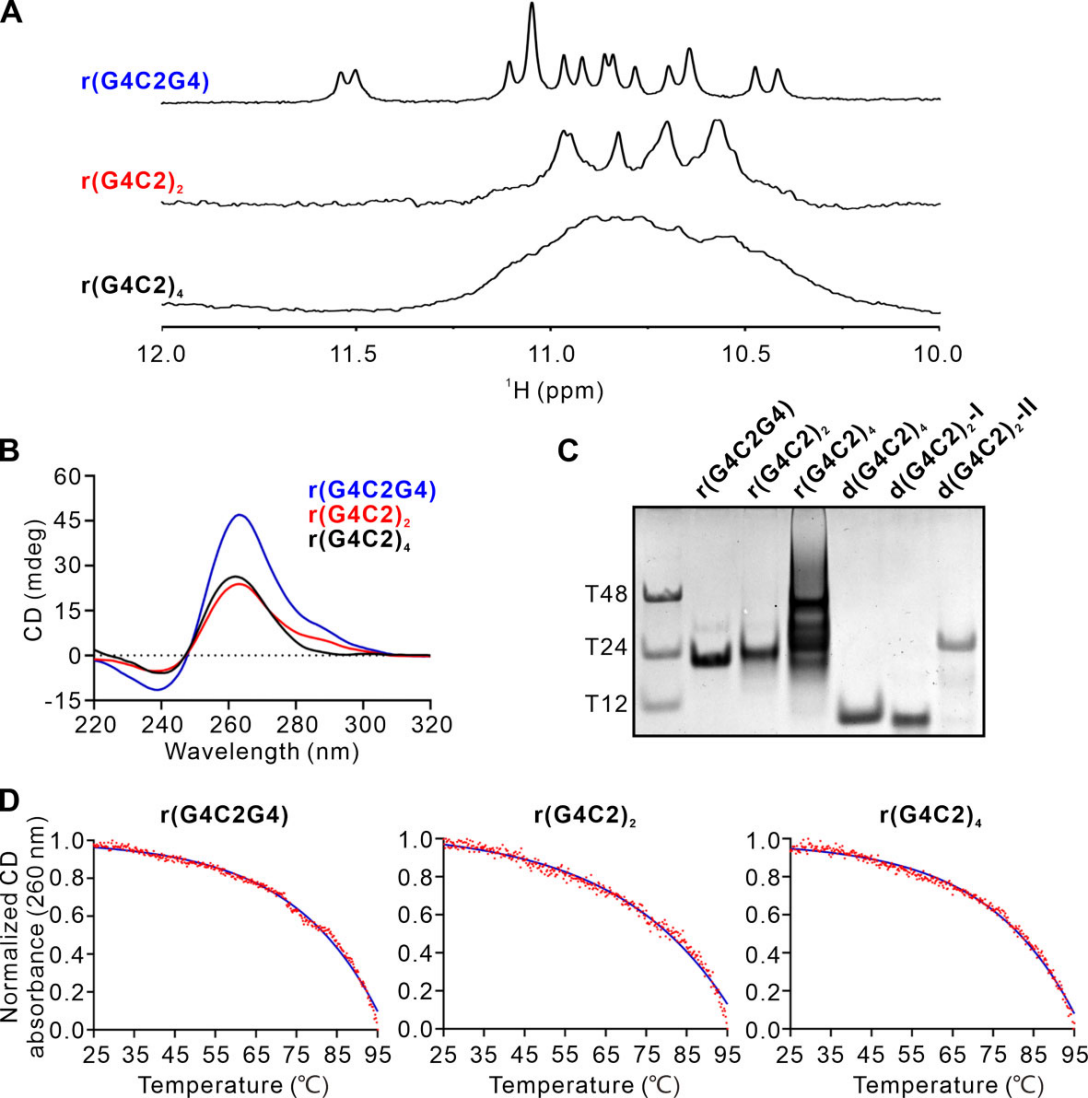
Characterization of r(G4C2)_n_ in K^+^ solution. (**A**) The imino region of 1D ^1^H NMR spectra of r(G4C2G4), r(G4C2)_2_ and r(G4C2)_4_ in the buffer containing 70 mM KCl, 20 mM potassium phosphate (pH 7.0) recorded at 25°C on 800 MHz NMR spectrometer. (**B**) CD spectra of r(G4C2G4), r(G4C2)_2_ and r(G4C2)_4_ in the buffer containing 70 mM KCl, 20 mM potassium phosphate (pH7.0) recorded at 25°C. (**C**) Electrophoretic mobility in non-denaturing 20% PAGE of r(G4C2G4), r(G4C2)_2_ and r(G4C2)_4_ with the concentration of 100 μM. The references: d(G4C2)_4_ (d[(GGGGCC)_4_]), a monomeric four-layer antiparallel G-quadruplex; d(G4C2)_2_-Form I, a dimeric three-layer hybrid G-quadruplex; d(G4C2)_2_-Form II, a tetrameric eight-layer parallel G-quadruplex formed by d(G4C2)_2_ (37,43). (**D**) CD melting curves of r(G4C2G4), r(G4C2)_2_ and r(G4C2)_4_ in the buffer containing 70 mM KCl, 20 mM potassium phosphate (pH7.0). Data were fit by the Boltzmann sigmoid equation (GraphPad Prism).

It is well known that *C9orf72* HRE G4C2 DNA repeats can form a mixture of G-quadruplex conformations in the K^+^ solution (37). To study the structure of r(G4C2)_*n*_, we first screened *C9orf72* HRE RNA sequences with various lengths, including r(G4C2G4), r(G4C2)_2_ and r(G4C2)_4_. Not surprisingly, the 1D ^1^H NMR spectra shows that r(G4C2G4), r(G4C2)_2_ and r(G4C2)_4_ can fold into G-quadruplexes in the K^+^ solution, indicated by the typical imino proton peaks at 10–12 ppm suggesting the formation of G-tetrad (Figure 1A). Furthermore, the circular dichroism (CD) spectroscopy, characterized by a dominant positive peak at ∼260 nm and a negative peak at ∼240 nm, clearly indicates a parallel G-quadruplex fold adopted by these RNA sequences (Figure 1B).

To probe the molecular size and homogeneity of r(G4C2)_*n*_ in K^+^ solution, we performed native polyacrylamide gel electrophoresis (PAGE) experiment using references including DNA oligonucleotides dT12, dT24 and dT48, d(G4C2)_4_ adopting a monomeric 24bp four-layer antiparallel G-quadruplex (42), d(G4C2)_2_-Form I (a dimeric three-layer hybrid G-quadruplex) and d(G4C2)_2_-Form II (a tetrameric eight-layer parallel G-quadruplex) formed by d(G4C2)_2_ (37,43). As shown in Figure 1C, the monomeric G-quadruplex formed by d(G4C2)_4_ and the dimeric hybrid G-quadruplex of d(G4C2)_2_-Form I migrated similarly as dT12. However, the migration of r(G4C2)_2_ in K^+^ solution is slower than dT12 and comparable to dT24 and d(G4C2)_2_-Form II which forms a tetrameric eight-layer parallel G-quadruplex (36), indicating formation of multimeric structures and potentially a tetrameric G-quadruplex.

Besides, the 1D ^1^H NMR spectra of r(G4C2G4) and r(G4C2)_2_ display an excellent dispersion at 10–12 ppm (Figure 1A), indicating a predominant G-quadruplex conformation formed by r(G4C2G4) and r(G4C2)_2_ in solution, which is also supported by one major band observed in the native PAGE experiment (Figure 1C). Whereas, r(G4C2)_4_ forms a mixture of multiple G-quadruplex structures indicated by several bands observed in the native PAGE experiment result (Figure 1C), which is consistent with the broad profile displayed at 10–12 ppm in the 1D ^1^H NMR spectrum (Figure 1A).

Furthermore, the size-exclusion chromatography coupled with multi-angle light scattering (SEC-MALS) analysis showed that the molecular weight of r(G4C2)_2_ is 14.7 ± 0.6 kDa, which is close to the theoretical molecular weight of tetramer (14.9 kDa) (Supplementary Figure S1). Altogether, both native PAGE and SEC-MALS results suggest a predominant tetrameric G-quadruplex adopted by r(G4C2)_2_ in solution, which is consistent with the crystal structure reported here (see below).

### Stability of r(G4C2)_*n*_ investigated by CD melting experiments

The thermal stability of the G-quadruplexes formed by r(G4C2G4), r(G4C2)_2_ and r(G4C2)_4_ was examined by CD melting experiments in buffer of 20 mM potassium phosphate (pH 7.0) with 35 and 70 mM KCl, respectively. The CD spectrum showed that r(G4C2G4), r(G4C2)_2_ and r(G4C2)_4_ adopt parallel G-quadruplexes in both 35mM and 70mM KCl solution (Supplementary Figure S2 and Figure 1D). The melting profiles of r(G4C2G4), r(G4C2)_2_ and r(G4C2)_4_ show no obvious transition (Figure 1D), indicating the conformational heterogeneity of these three RNAs, although only one band is observed for r(G4C2)_2_ by Native PAGE (Figure 1C). Interestingly, the *T*_m_ values showed that the thermostability of r(G4C2)_2_ increased in higher salt concentration (Table 1). Altogether these data indicate that r(G4C2G4), r(G4C2)_2_ and r(G4C2)_4_ form very stable G-quadruplexes, r(G4C2)_2_ adopts a unique conformation in comparison with r(G4C2G4) and r(G4C2)_4_.

### Overall structure of G-quadruplex formed by r(G4C2)_2_ in K^+^ solution

**Figure 2.**
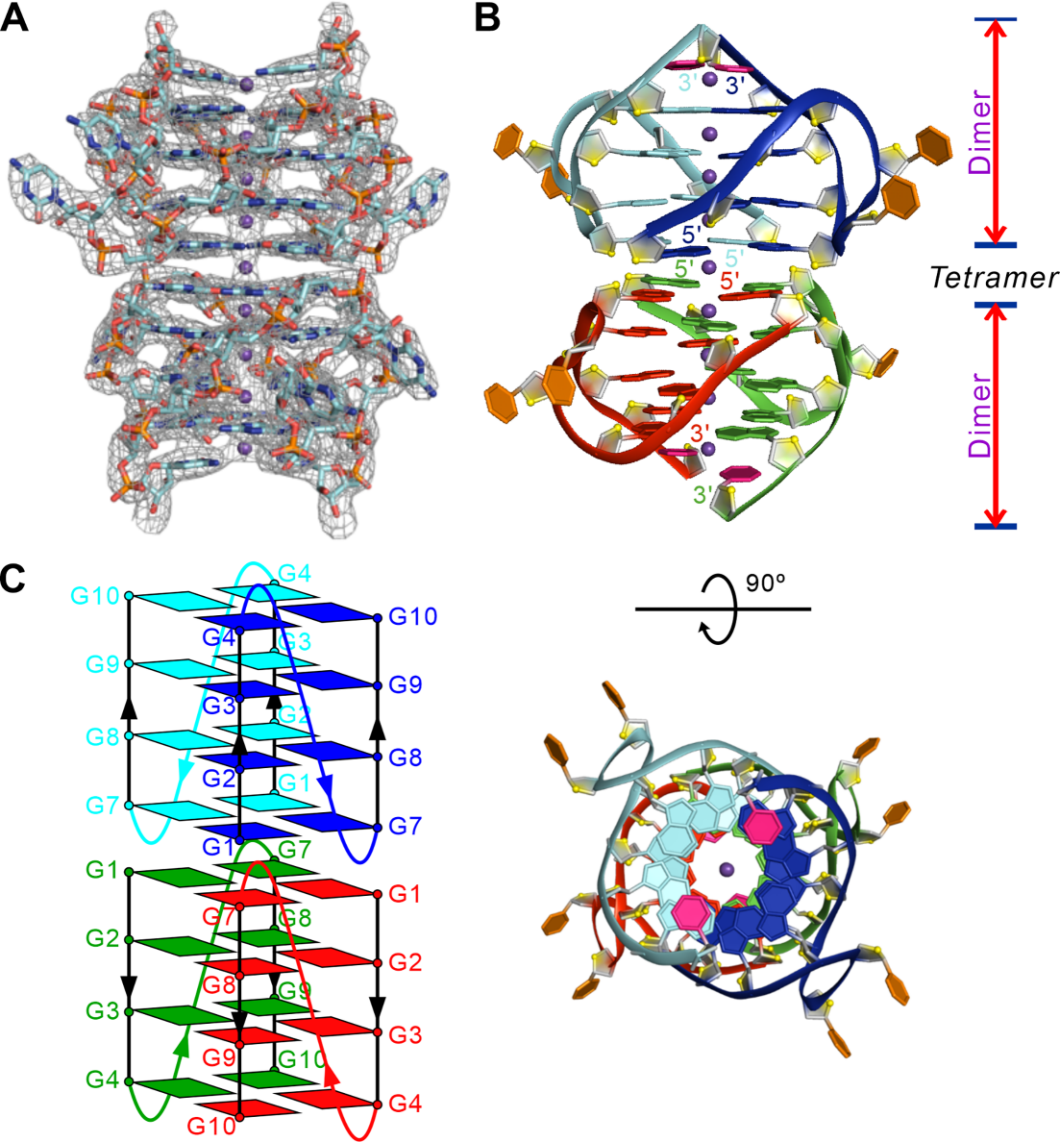
Overall structure of G-quadruplex formed by r(G4C2)_2_ in K^+^ solution. (**A**) The electron density map of the final 2mFo – DFc map contoured at 1.0 σ for r(G4C2)_2_. (**B**) Cartoon representation of tetrameric G-quadruplex formed by r(G4C2)_2_. Each dimeric block is stacked to form a tetrameric G-quadruplex via different 5′-arrangments and stabilized by K^+^ (purple sphere). Each molecule, d(G4C2)_2_, is shown as red, green, blue and cyan in the tetrameric G-quadruplex. O4' oxygens are in yellow. (**C**) Schematic representation of topology adopted by r(G4C2)_2_.

As r(G4C2)_2_ represents homogenous G-quadruplex structures and adopts stable conformation, which indicated by the biophysical characterization, various crystallization conditions were screened. Finally, we successfully solved the crystal structure of r(G4C2)_2_ in the space group of *P*6_1_22 with the resolution of 2.96 Å (Table 2). There are two chains of r(G4C2)_2_ oligonucleotides in the asymmetric unit of the crystal (Supplementary Figure S3A). The electron densities are well defined nearly all residues except the last cytosine residue (Figure 2A).

**Table 2.** Crystallographic data collection and refinement statistics

**Data collection**	r(G4C2)_2_
Space group	*P*6_1_22
Wavelength (Å)	0.97891
Unit cell parameters	*a* = *b* = 62.971 Å, *c* = 129.227 Å, α=β=90° γ=120°
Resolution range (Å)	50–2.956 (3.00–2.956)
No. of unique reflections	3568 (172)
Redundancy	22.5 (24.8)
I/σ	22.46 (1.18)
Completeness (%)	99.9 (100.0)
*R*_merge_^a^ (%)	15.0 (176.8)
**Structure refinement**	
Resolution (Å)	2.956
*R*_work_^b^ (%)	26.11
*R*_free_^c^ (%)	29.99
RMSD bonds (Å)	0.006
RMSD angles (°)	2.003
Average *B* factor (Å^2^)	67.7
No. of atoms	
RNA	480
Water	1
Ion	9
*B* factor (Å^2^)	
RNA	67.8
Water	29.8
Ion	65.9

Numbers in parentheses represent the values for the highest-resolution shell.

^a^ *R*_merge_ = Σ|*I*_i_ – |/Σ*I*_i_, where *I*_i_ is the intensity of measured reflection and is the mean intensity of all symmetry-related reflections.

^b^ *R*_work_ = Σ_W_||*F*_calc_| – |*F*_obs_||/Σ|*F*_obs_|, where *F*_obs_ and *F*_calc_ are observed and calculated structure factors. *W* is working dataset of about 95% of the total unique reflections randomly chosen and used for refinement.

^c^ *R*_free_ = Σ_T_||*F*_calc_| – |*F*_obs_||/Σ|*F*_obs_|, where *T* is a test dataset of about 5% of the total unique reflections randomly chosen and set aside prior to refinement.

**Figure 3.**
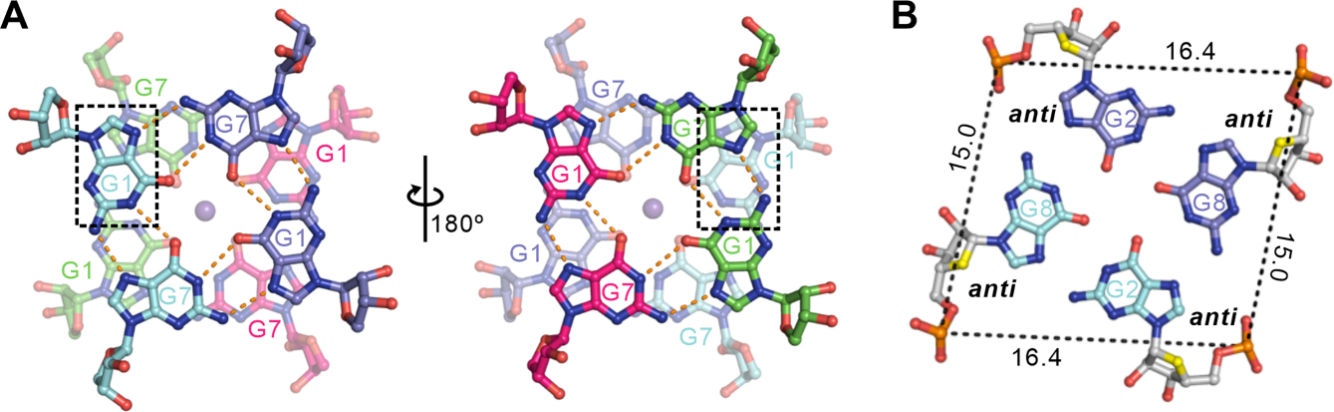
The detailed stacking mode and K^+^ ions in the structure of r(G4C2)_2_. (**A**) The G-tetrad base stacking mode at the interface of two dimeric block in the tetrameric G-quadruplex crystal structure of r(G4C2)_2_. The hydrogen bonds are represented by dash orange lines. (**B**) The G-tetrad, G2·G8·G2·G8. Average groove width values are indicated by phosphate–phosphate distances shown as dashed lines.

Intriguingly, chains A/B and their crystallographically symmetric molecules (chains A’/B’) form a parallel-stranded dimeric G-quadruplex unit (i.e. chains A/A’, chains B/B’), which is composed of four G-tetrads connected by two CC double-chain-reversal loops (Figure 2B and Supplementary Figure S3B). The dimeric G-quadruplex unit co-axially stacks on the other crystallographically symmetric dimeric G-quadruplex unit in a 5′-to-5′ arrangement, resulting in a tetrameric eight-layer G-quadruplex through π–π interactions (Figure 2B and C). The G1 base in one dimeric block stacks (i.e. chains A/B) with the G7 base in the opposite dimeric block (i.e. chains A’/B’) (Figure 2C and 3A).

The same stacking mode is observed in the G-quadruplex structure formed by *C9orf72* DNA HRE, d(G4C2)_2_, termed as Form-1/7 (36). In particular, the crystal structure of r(G4C2)_2_ showed here is almost identical to the parallel propeller-type tetrameric G-quadruplex structure of d(G4C2)_2_ with RMSD ∼2.42Å, composed of two identical dimeric G-quadruplex co-axially stacking via a 5′-to-5′ arrangement (Supplementary Figure S4). A recent survey showed that the experimentally observed base stacking geometries at the interface of stacked G-quadruplexes are classified into four modes, ‘Partial 6-ring’, ‘6-ring’, ‘5/6-ring’ and ‘5-ring’, the relative position of neighboring Guanines of G-core as shown in Supplementary Figure S5 (44). Interestingly, the base stacking mode at the interface is ‘Partial 5-ring’ in the crystal structure of r(G4C2)_2_, which is the first experimentally observed (Figure 3A). Each dimeric G-quadruplex has four medium grooves with widths of 15.0/16.4 Å (Figure 3B).

The hydrogen-bond directionalities (donor to acceptor) of the four G-tetrads in each dimeric block are clockwise (G1→G7→G1'→G7', G2→G8→G2'→G8', G3→G9→G3'→G9' and G4→G10→G4'→G10' with the prime (') signifying the bases belong to separate oligonucleotide strands in the same dimeric block) with all Hoogsteen N1–O6 and N2–N7 hydrogen bonds intact (Figure 2). The glycosidic conformations of all bases in each G-quadruplex are *anti* (Figure 3B).

### The K^+^ in the structure of r(G4C2)_2_

Notably, the electron density was well defined for the nine equal-spaced K^+^ ions lying along the axis within the central core of the tetrameric G-quadruplex including well-defined central channel potassium ions located in the interface between the two dimeric blocks (Figure 2A, B and Supplementary Figure S6). Seven K^+^ ions locate in the G-core of the tetramer coordinating to eight neighbouring guanine O6 atoms at a distance of ∼2.8 Å leading to an anti-prismatic coordination environment. Interestingly, another two K^+^ ions coordinate to four neighbouring guanine O6 atoms of the outer G-tetrad at a distance of ∼3.2 Å and bridge the two cytosine in the 3′-end (Supplementary Figure S6).

In particular, the K^+^ connecting the two dimeric units may result in the absence of clear transition in the melting profile of r(G4C2)_2_, which potentially can be affected by the K^+^ concentration indicated by CD melting experiment (Figure 1D and Table 1).

### The cytosine conformations in the structure of r(G4C2)_2_

**Figure 4.**
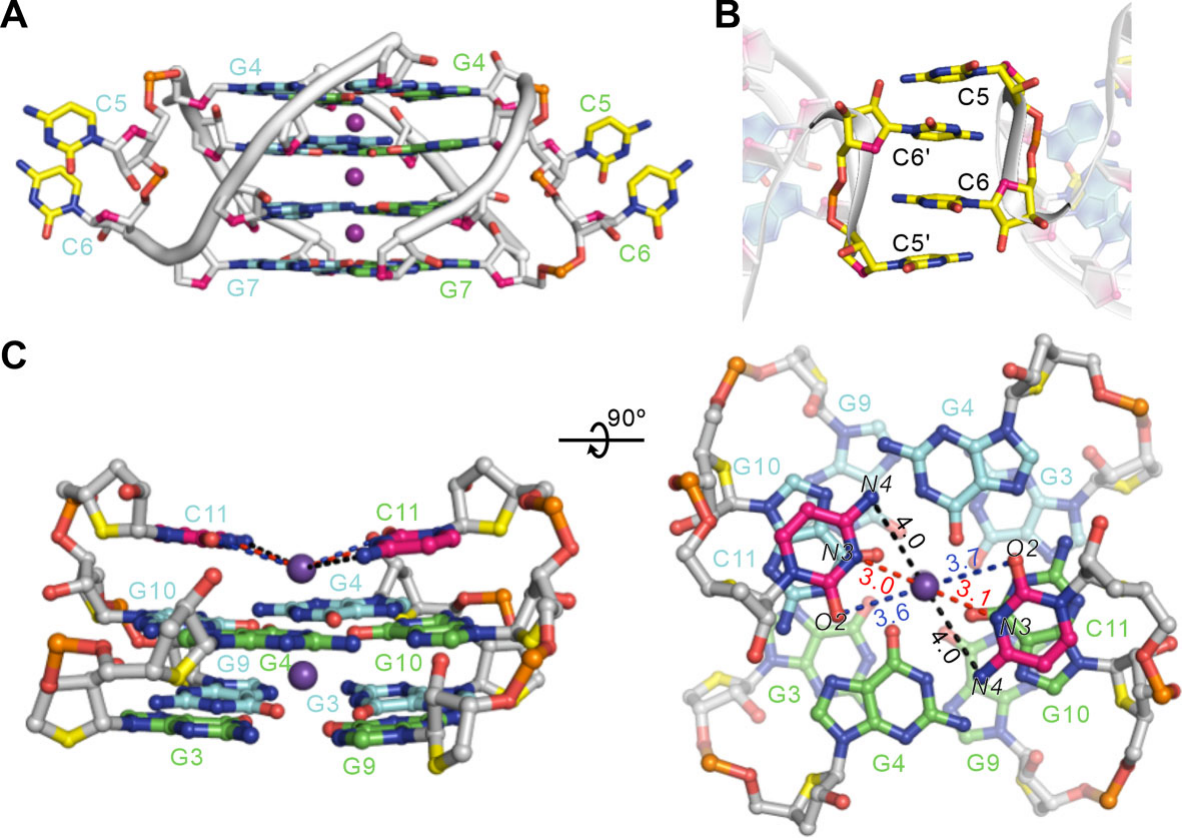
Detailed conformations of cytosines in the tetrameric G-quadruplex formed by r(G4C2)_2_. (**A**)The conformation of propeller loop, C5 and C6, in the dimeric G-quadruplex. (**B**) Intermolecular π−π packing interactions for C5 and C6 bases observed in the in the unit cell. The prime (′) notation signifies that the two bases belong to separate oligonucleotide strands. (**C**) The conformation of the C11 base located at the 3′-end and its interaction K^+^ ion. The distances between K^+^ ion and the O2, N3 and N4 of C11 are represented by dash lines.

In the dimeric block of r(G4C2)_2_ G-quadruplex, the four G-tetrads are connected by two propeller loops composed of C5 and C6 bases (Figure 2). The C5 and C6 almost parallel without stacking interaction and protrude out (Figure 3A and Supplementary Figure S7). Interestingly, additional intermolecular π−π packing interactions for C5 and C6 bases were observed as shown in Figure 4B, which bridges the tetrameric G-quadruplex in crystal unit cell.

For the two cytosine bases located at 3′ end of each stand, only C11 base was well defined in the electron density and stacks on G-tetrad of the dimeric block. The C12 cannot be observed in the electron density, indicating the flexibility of the 3′-end (Supplementary Figure S7). Interestingly, two additional K^+^ ions were observed in the same plane with C11 and C12 bases. The angle between the N3 atom of C11, K^+^ ion and the N3 atom of C11' is∼ 144° , indicating the π−π packing interactions between the C11 bases and G-tetrad (Figure 4C). Intriguingly, the distance analysis shows that no C·C base pair was observed between the C11 bases of the neighbouring oligonucleotides. Notably, the distance between the N3 atom of C11 base and K^+^ ion is ∼3.0Å. However, the distances between the O2 atom and N4 atom and K^+^ ion is ∼3.6 and ∼4.0 Å, respectively (Figure 4C). These distances indicate that K^+^ ion play an important contribution in stabilizing the C11 conformations.

## Discussion

Numerous studies have been made on the G-quadruplex structures formed by *C9orf72* DNA (G4C2)_*n*_ (36,37,42,45,46). However, no RNA G-quadruplex structure formed by RNA (G4C2)_*n*_ has been reported. Here, for the first time, we determined the crystal structure of r(G4C2)_2_, a parallel eight-layer G-quadruplex composed of two dimeric G-quadruplex via 5′-to-5′ stacking.

Recently, we reported the G-quadruplex structure formed by two DNA G4C2 repeats, d(G4C2)_2_ (36). Notably, the G-core of r(G4C2)_2_ is nearly identical to that of d(G4C2)_2_, with a root-mean-square deviation (RMSD) of approximately 2.42 Å (Supplementary Figure S4). However, the average width of medium grooves formed by the G-core in r(G4C2)_2_ is narrower than that in d(G4C2)_2_ by about 0.6–0.9 Å (Supplementary Table S1). Further analysis indicates that this narrowing may be attributed to the 2′OH groups present in RNA, which result in a more constricted and shallower groove compared to DNA (Supplementary Figure S8). Strikingly, the average groove width of G1·G7·G1·G7 in d(G4C2)_2_ appears to be approximately 1.6 Å wider than that in r(G4C2)_2_ (Supplementary Table S1). This discrepancy could be due to the additional 5′-to-5′ stacking interactions, which are not counterbalanced by the 2′OH groups as in the RNA structure (Supplementary Figure S8). Therefore, the presence of the 2′OH groups and the 5′-to-5′ stacking mode are likely key factors contributing to the observed difference in groove width between the DNA and RNA G-quadruplexes, which could be important structure features for RNA binding protein to recognize.

Although both the C5 and C6 bases of r(G4C2)_2_ protrude away from the G-core, the C6 base of d(G4C2)_2_ is inserted into the medium groove of the G-core (Supplementary Figure S9). This insertion is further stabilized by a hydrogen bond as shown in our previous study (36). The specific positioning of the C6 base in d(G4C2)_2_ could potentially account for the broader groove observed in d(G4C2)_2_ compared to r(G4C2)_2_. Additionally, the C11 and C12 bases at the 3′ end of d(G4C2)_2_ are located outside the G-core and may not contribute to the stability of the G-quadruplex. This contrasts with the C11 base in r(G4C2)_2_, which is observed to stack on the G-core (Supplementary Figure S9). Collectively, the unique conformations observed in our study, particularly the differences between G4C2 DNA and RNA in the loop region and groove size, including the impact of the 2′OH groups in the RNA G-quadruplex, are significant for both targeted drug design and understanding the pathogenic mechanisms of ALS/FTD.

A search of a quadruplex structure database (ONQUADRO: https://onquadro.cs.put.poznan.pl/home) yields 57 G-quadruplex structures, formed by RNA alone or in complex with proteins, which have been experimentally determined and deposited in PDB (https://www.rcsb.org) (47). Upon visual examination of the 57 structures, four dimeric G-quadruplexes containing four G-tetrad layers, formed by two unique sequences, show a similar 5′-to-5′ stacking with r(G4C2)_2_ structure (PDB ID: 2RQJ, 2RSK, 2RU7 and 1MY9). In particular, there are six tetrameric G-quadruplexes containing eight G-tetrad layers, formed by r(UGGGGU) in the presence of K^+^ and/or Sr^2+^/Ba^2+^, also display a similar 5′-to-5′ stacking (PDB ID:1J8G, 1RAU, 4RJ1, 4RKV, 4RNE and 4XK0). There are also another eight G-quadruplex structures containing four G-tetrad layers and 39 G-quadruplexes composed of two/three G-tetrad layers without 5′-to-5′ stacking mode. The analysis indicates that 5′-to-5′ stacking, potentially including 3′-to-3′ stacking, plays a crucial role in stabilizing RNA G-quadruplex, particularly in higher-order G-quadruplex structure *in vivo*.

**Figure 5.**
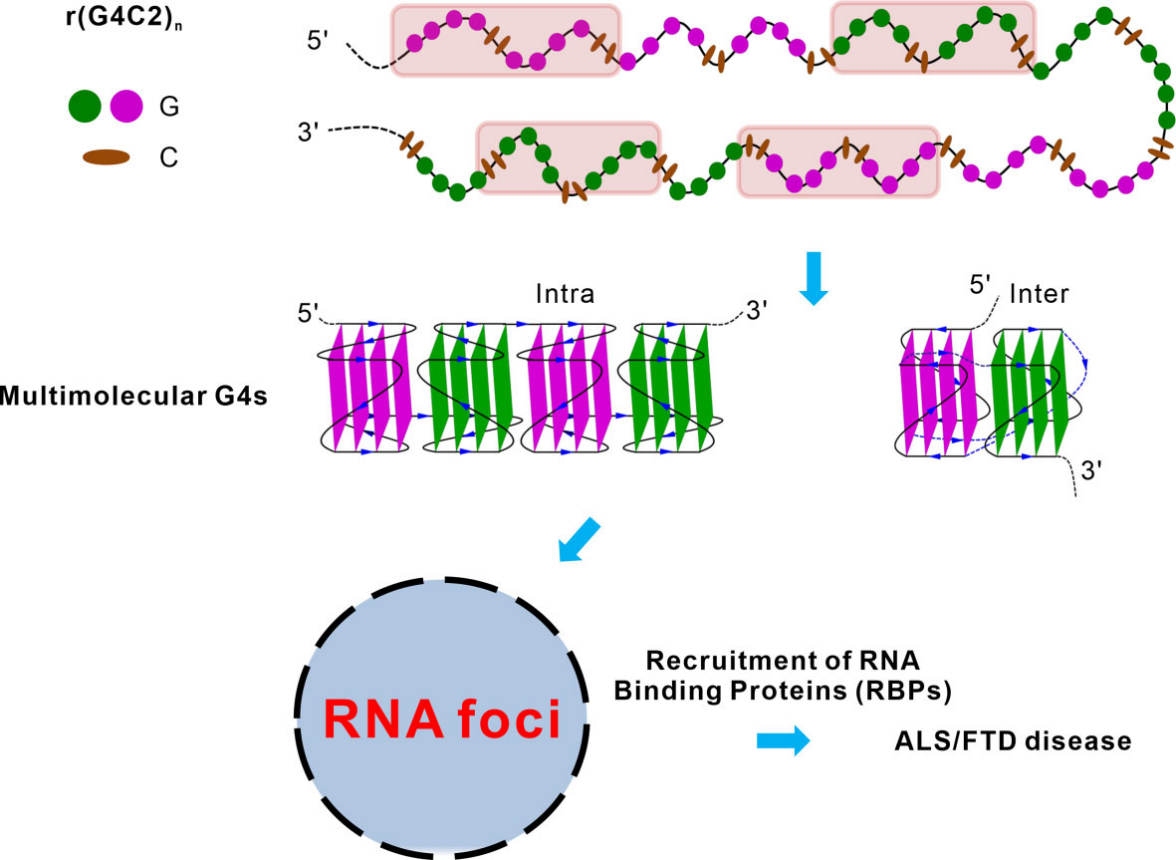
Schematic diagram showing the formation of multimeric G-quadruplex by the r(G4C2)n. The sequential (G4C2)_4_ can form intramolecular G-quadruplexes and the (G4C2)_2_ in rectangular colored by pink can form intermolecular G-quadruplexes. Both intramolecular and intermolecular G-quadruplexes can further pack together to form multimeric G-quadruplexes, leading to the formation of RNA foci which recruit RNA binding proteins (RBPs) to cause ALS/FTD.

As shown in Figure 5, RNA G4C2 HRE is proposed to adopt intramolecular four-layer parallel G-quadruplexes, which can sequentially stack together via 5′-to-5′ stacking to from compact-stacking higher-order quadruplex structures. Moreover, four neighbouring r(G4C2)_2_ repeats locating in the different positions of the same chain can form intermolecular eight-layer parallel G-quadruplex structures as reported here, in which two four-layer parallel G-quadruplex units are connected by long G4C2 repeats. These multimolecular G-quadruplexes, both intra- and inter- molecules, pack together to form RNA foci (Figure 5). Although further experimental data is needed to validate the model we hypothesized, our structure highlights the possible features required for studying the higher-order quadruplex structure of r(G4C2)_*n*_*in vivo* and the formation of toxic RNA foci.

It was well-known that the transcribed r(G4C2)_*n*_ observed in ALS/FTD patients forms RNA foci that recruit RNA binding proteins (RBPs), consequently leading to impairment of RBPs’ function that triggers cellular cytotoxicity (13,23,48) (Figure 5). In particular, this process involves the spontaneous liquid–liquid phase separation (LLPS) of r(G4C2)_*n*_ (27,49,50), which depends on the multivalent intermolecular interactions (51,52). Based on the tetrameric structure of r(G4C2)_2_ reported here, it is reasonable to speculate that G-quadruplex formed by r(G4C2)_*n*_ is also multimeric *in vivo*, intramolecular four-layer and intermolecular eight-layer parallel G-quadruplex structures which stack together via 5′-to-5′ stacking mode. These multimeric G-quadruplexes aid long-range interactions and provides the multivalent intermolecular interactions to promote formation of the phase separation of G4C2 repeat-containing RNA. Furthermore, the phase separation of RNA occurs when the valence increases due to the growing repeat number. The multimeric structures formed by r(G4C2)_*n*_ via 5′-to-5′ stacking observed in our structure could explain why ALS/FTD disease is triggered after the G4C2 repeat expansions reach a certain threshold of repeat number (26,27) and the formation of RNA granules which play a role in the pathogenesis of the diseases (49).

Consequently, the G-quadruplex structure of r(G4C2)_*n*_ is a highly prominent drug target for *C9orf72*-linked ALS/FTD. Currently, only three drugs, riluzole, edaravone and AMX0035, have been approved by FDA for the treatment of ALS (53–55). Unfortunately, these two drugs can only delay disease progression but not cure and importantly they do not specifically target r(G4C2)_*n*_. Recently, several small molecules have been discovered to recognize and stabilize the r(G4C2)n G-quadruplex structures, thereby inhibiting RNA foci formation (56,57) and/or preventing non-ATG translation (34). Another promising development is that a small molecule, TMPyP4, was characterized to be able to disrupt the G-quadruplex formation of r(G4C2)_8_, and ablate the interaction between the G-quadruplex and its binding proteins (58). However, structural mechanism of these small molecules stabilizing/disrupting r(G4C2)_*n*_ G-quadruplexes remains elusive which hinders the drug development for treatment of LAS/FTD. Therefore, our structure provides a structural basis to elucidate the pathological mechanism caused by r(G4C2)_n_ and particularly in the *ad hoc* design of novel lead compounds targeting r(G4C2)_*n*_ G-quadruplexes, marking an important step toward the development of targeted therapies for these debilitating neurodegenerative diseases.

## Supplementary Material

gkae473_Supplemental_File

## Data Availability

Atomic coordinates and structure factors for the reported crystal structures of r(G4C2)_2_ have been deposited with the Protein Data Bank under accession number 8X0S.
