# Supplementary material for: Crystal structure of a tetrameric RNA G-quadruplex formed by hexanucleotide repeat expansions of C9orf72 in ALS/FTD
Source: Nucleic Acids Res. 2024 Jun 11;52(13):7961–70. doi: 10.1093/nar/gkae473 (PMC11260476; doi:10.1093/nar/gkae473)

**Supporting Information**

**Crystal structure of a tetrameric RNA G-quadruplex formed by hexanucleotide repeat expansions of *C9orf72* in ALS/FTD**

Yanyan Geng^1, 2, †^, Changdong Liu^2, 3, †, *^, Naining Xu^2, 3, †^, Monica Ching Suen^2, 3^, Haitao Miao^2^, Yuanyuan Xie^4^, Bingchang Zhang^4^, Xueqin Chen^1^, Yuanjian Song^5^, Zhanxiang Wang^4^, Qixu Cai^6, *^, and Guang Zhu^2, 3, *^

^1^ Clinical Research Institute of the First Affiliated Hospital of Xiamen University, Fujian Key Laboratory of Brain Tumors Diagnosis and Precision Treatment, Xiamen Key Laboratory of Brain Center, the First Affiliated Hospital of Xiamen University, School of Medicine, Xiamen University, Xiamen, Fujian, China

^2^ Institute for Advanced Study and State Key Laboratory of Molecular Neuroscience, Division of Life Science, The Hong Kong University of Science and Technology, Clear Water Bay, Kowloon, Hong Kong SAR, China

^3^ HKUST Shenzhen Research Institute, Hi-Tech Park, Nanshan, Shenzhen, Guangdong, China

^4^ Department of Neurosurgery and Department of Neuroscience, Fujian Key Laboratory of Brain Tumors Diagnosis and Precision Treatment, Xiamen Key Laboratory of Brain Center, the First Affiliated Hospital of Xiamen University, School of Medicine, Xiamen University, Xiamen, Fujian, China

^5^ Jiangsu Key Laboratory of Brain Disease Bioinformation, Department of Genetics, Xuzhou Medical University, Xuzhou, Jiangsu, China

^6^ State Key Laboratory of Vaccines for Infectious Diseases, School of Public Health, Xiamen University, Xiamen, Fujian, China

* Correspondence to: [gzhu@ust.hk](mailto:gzhu@ust.hk); [qxcai@xmu.edu.cn](mailto:qxcai@xmu.edu.cn); [lcd@ust.hk](mailto:lcd@ust.hk);

^†^ The authors wish it to be known that, in their opinion, the first 2 authors should be regarded as joint First Authors.

**Figure S1. SEC-MALS assay of r(G4C2)_2_.** The result shows tetrameric G-quadruplex adopted by r(G4C2)_2_ in solution containing 70 mM KCl, 20 mM potassium phosphate (pH7.0). The fitted molecular weights are expressed as the best fitted values ± SE.


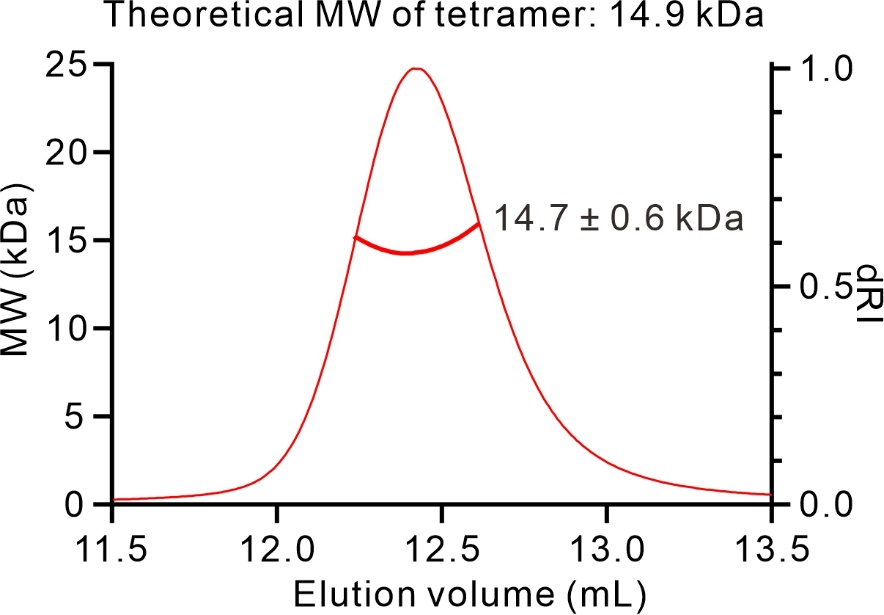


**Figure S2. CD characterization of r(G4C2)_2_** in the buffer containing 35 mM KCl, 20 mM potassium phosphate (pH7.0)**.** (A) CD spectra of rG4C2G4, r(G4C2)_2_ and r(G4C2)_4_ recorded at 25 °C. (B-D) CD melting curves of rG4C2G4 (B), r(G4C2)_2_ (C) and r(G4C2)_4_ (D). Data were fit by the Boltzmann sigmoid equation (GraphPad Prism).


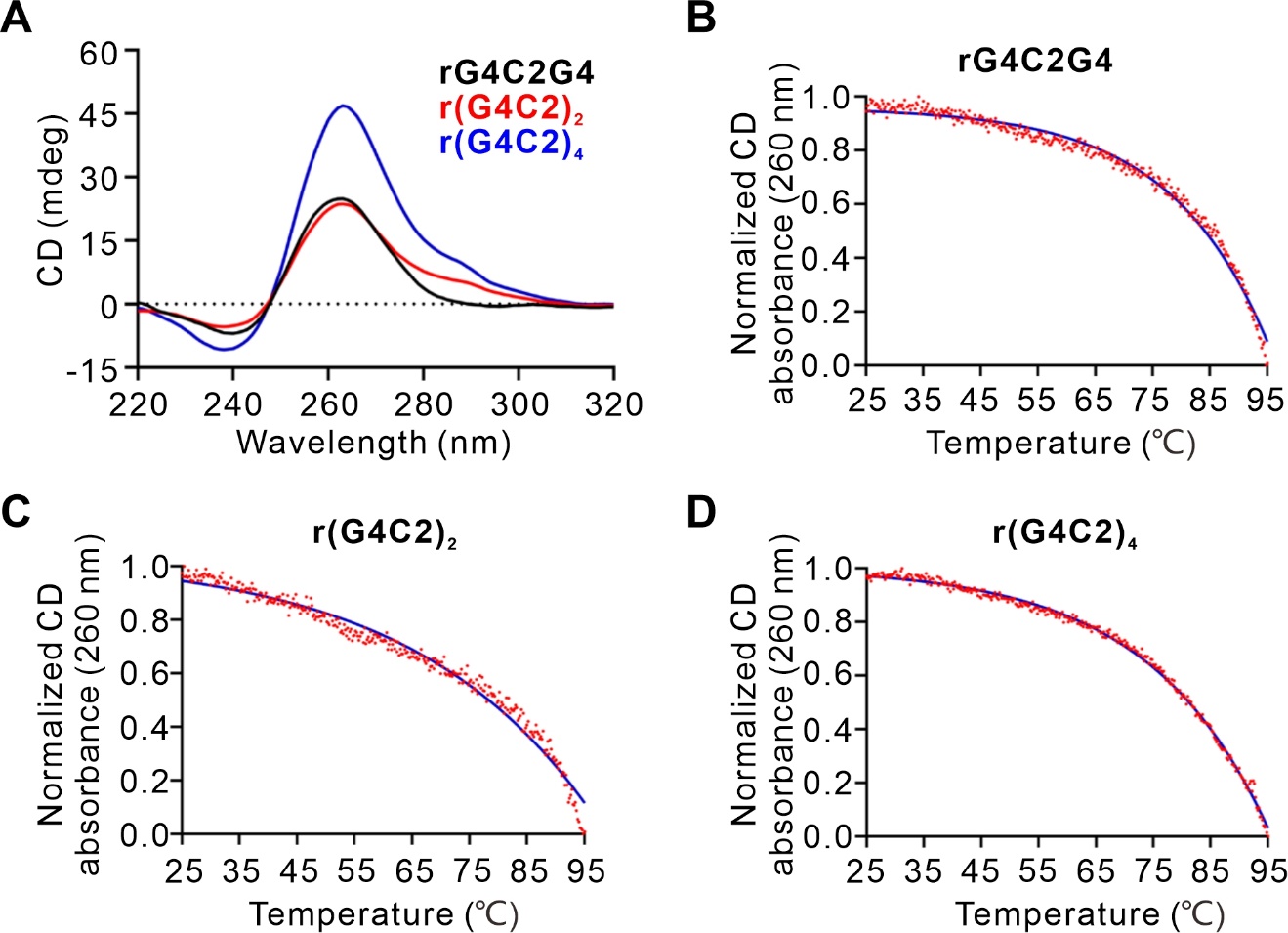


**Figure S3. The crystal structure of r(G4C2)_2_ in P6_1_22 space group.** (A) There are two chains of r(G4C2)_2_ in an asymmetric unit of crystal. (B) Crystal packing of r(G4C2)_2_. The tetrameric G-quadruplex is indicated by dashed rectangles.


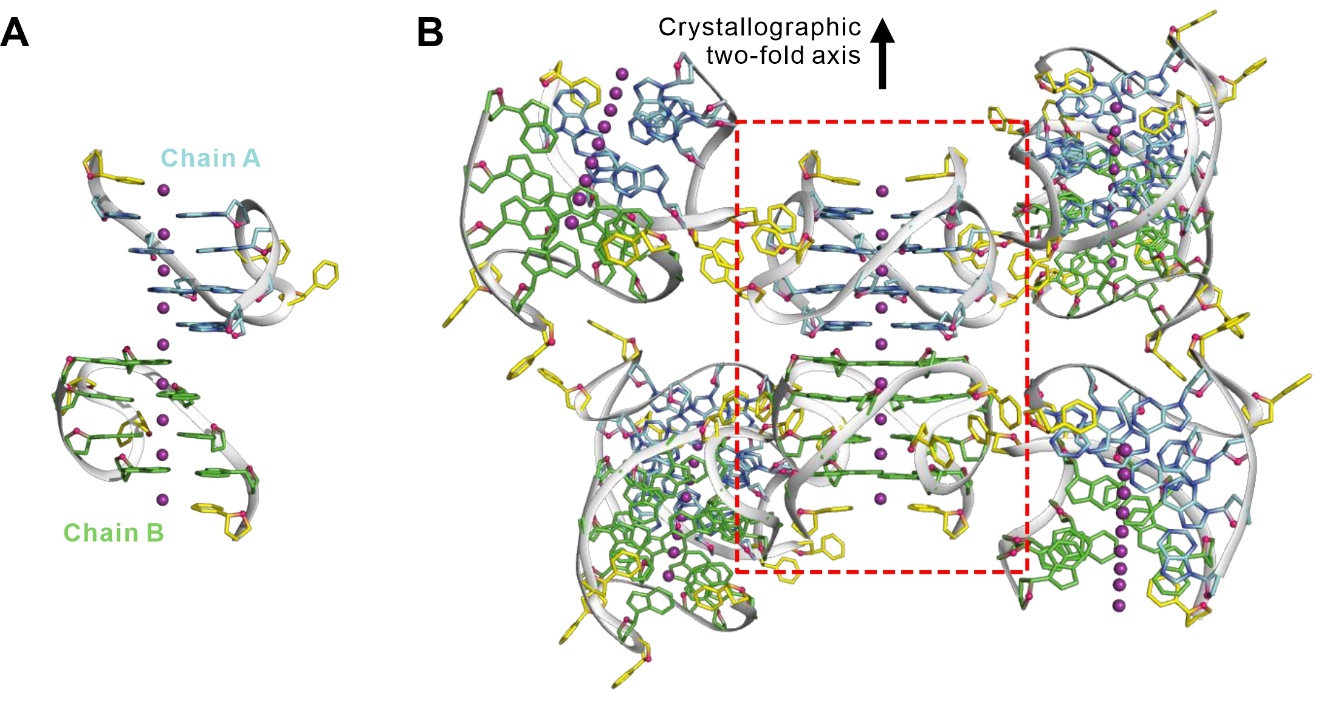


**Figure S4**. The overlaid structure of r(G4C2)_2_ (yellow) and d(G4C2)_2_-Form1/7 (blue).


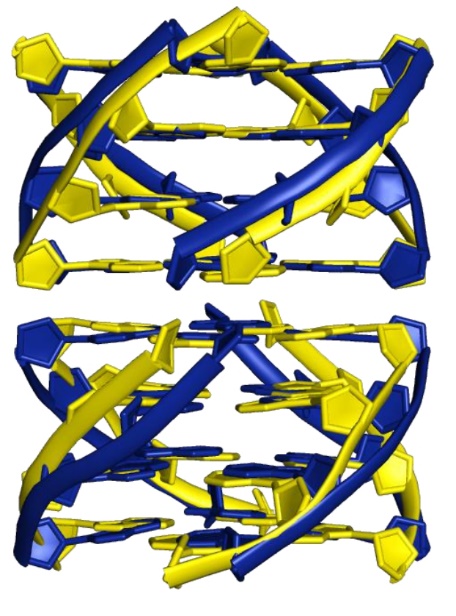


**Figure S5**. Illustrative examples of core base stacking modes. (A) ‘Partial 6-ring’, (B) ‘6-ring’, (C) ‘5/6-ring’, (D) ‘5-ring’.


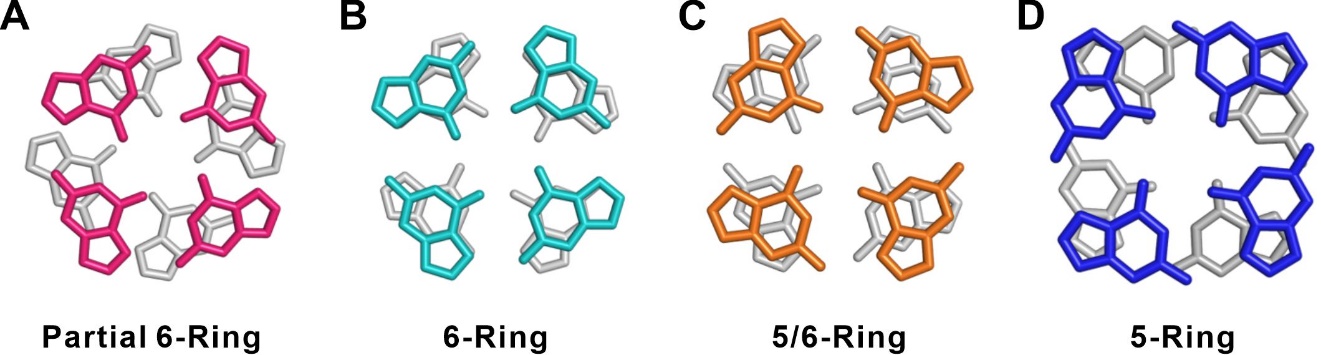


**Figure S6**. Expanded view of the environment of the K^+^ ions in the tetrameric G-quadruplex formed by r(G4C2)_2_, with the bonds between K^+^ and oxygen atom shown as dash black lines.


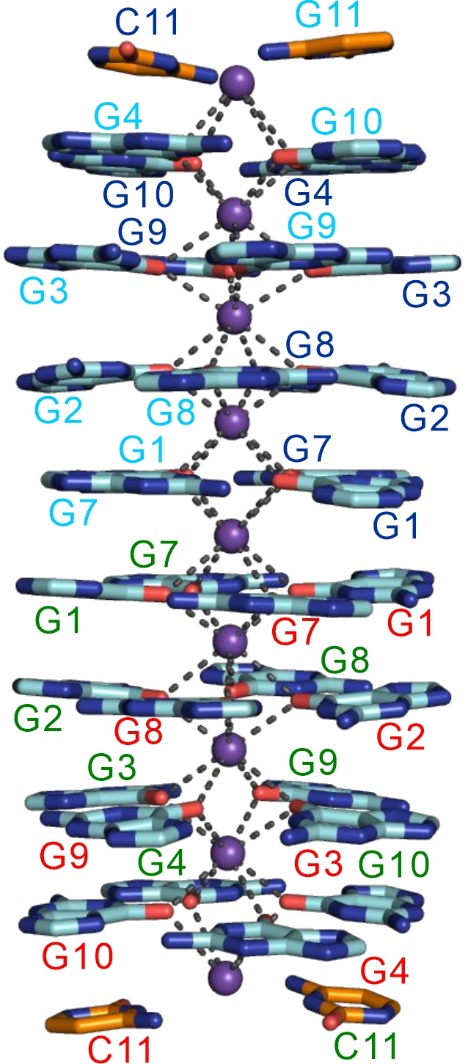


**Figure S7. The side view and top view of the cytosines, C5, C6, C11 and C12 bases, in the crystal structure of r(G4C2)_2_.** The G-tetrad core of the tetrameric G-quadruplex is shown in surface mode with the atoms of phosphate and phosphate oxygen colored in red.


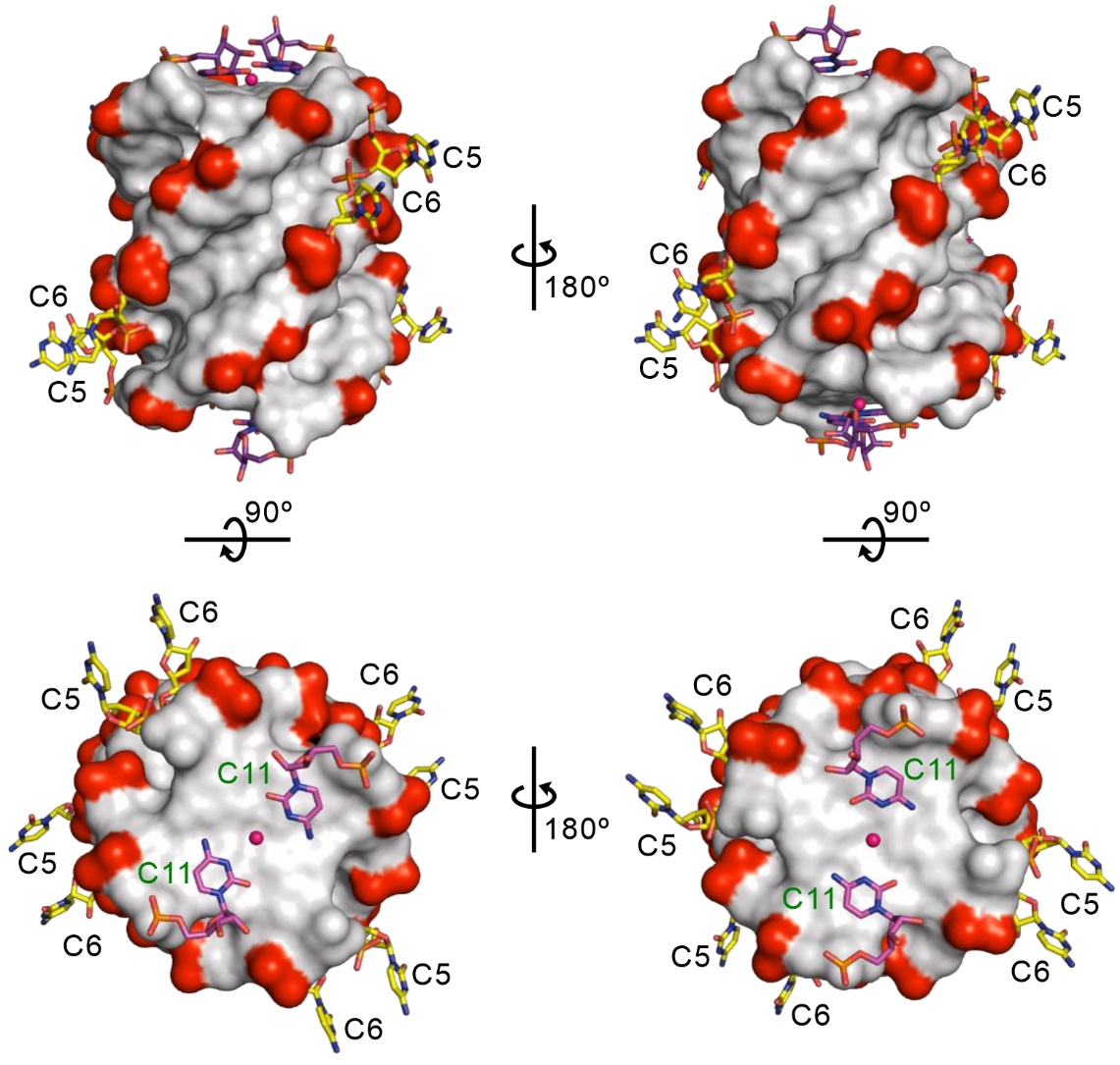


**Figure S8. The surface of G-core of r(G4C2)_2_ reported here and d(G4C2)_2_ (PDB:7ECH).** The G-core of (A) r(G4C2)_2_, (B) d(G4C2)_2_ and (C) the overlay of (A) and (B). The dashed rectangular indicates the 5'-to-5' stacking interface. The 2'OH group of r(G4C2)_2_ is colored by red.


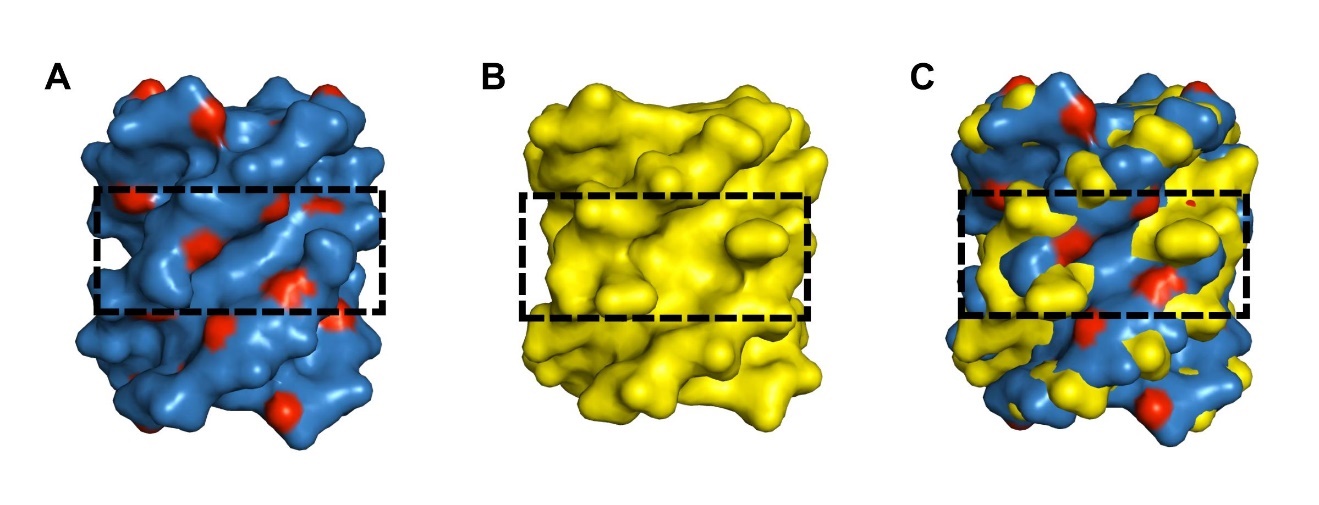


**Figure S9. The comparison of the CC conformation observed in r(G4C2)_2_ reported here and d(G4C2)_2_ (PDB:7ECH).** (A) The overlay of r(G4C2)_2_ with d(G4C2)_2_ in (A) cartoon and (B) surface mode. In (A), the G-core is colored by gray. In (B), the G-core shown in surface mode is colored by pink in RNA and lime in DNA, respectively. The 2'OH group of r(G4C2)_2_ is colored by red. The CC bases are colored by hotpink in RNA and orange in DNA, respectively.


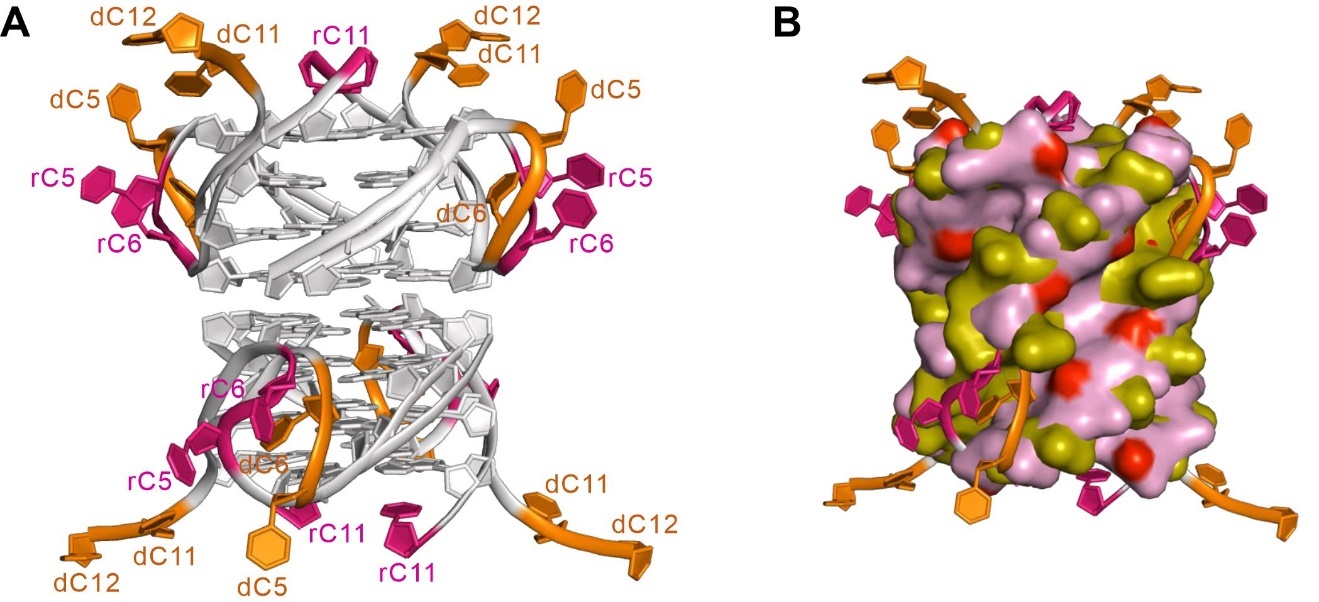


**Table S1** **The width of four medium grooves observed in structure of r(G4C2)_2_ reported here and d(G4C2)_2_ (PDB:7ECH).** M1, M2, M3 and M4 indicates medium groove 1, medium groove 2, medium groove 3 and medium groove 4. The average groove width values are indicated by phosphate-phosphate distances in each G-tetrad shown as dashed lines and M1-4 are defined in the bottom of figure.

|  | **r(G4C2)_2_** | | | | **d(G4C2)_2_** | | | |
| --- | --- | --- | --- | --- | --- | --- | --- | --- |
|  | M1 | M2 | M3 | M4 | M1 | M2 | M3 | M4 |
| G1·G7·G1·G7* | 15.1Å | 16.1Å | 15.1Å | 16.1Å | 16.7Å | 17.7Å | 16.7Å | 17.7Å |
| G2·G8·G2·G8 | 16.4Å | 15.0Å | 16.4Å | 15.0Å | 17.3Å | 15.7Å | 17.3Å | 15.7Å |
| G3·G9·G3·G9 | 15.8Å | 15.4Å | 15.8Å | 15.4Å | 16.4Å | 16.2Å | 16.4Å | 16.2Å |
| G4·G10·G4·G10 | 15.7Å | 15.4Å | 15.7Å | 15.4Å | 16.4Å | 16.3Å | 16.4Å | 16.3Å |

*As for the first G base at the 5' end, G1, misses the phosphate atom, which is replaced by C5' atom in calculation of the average groove width.


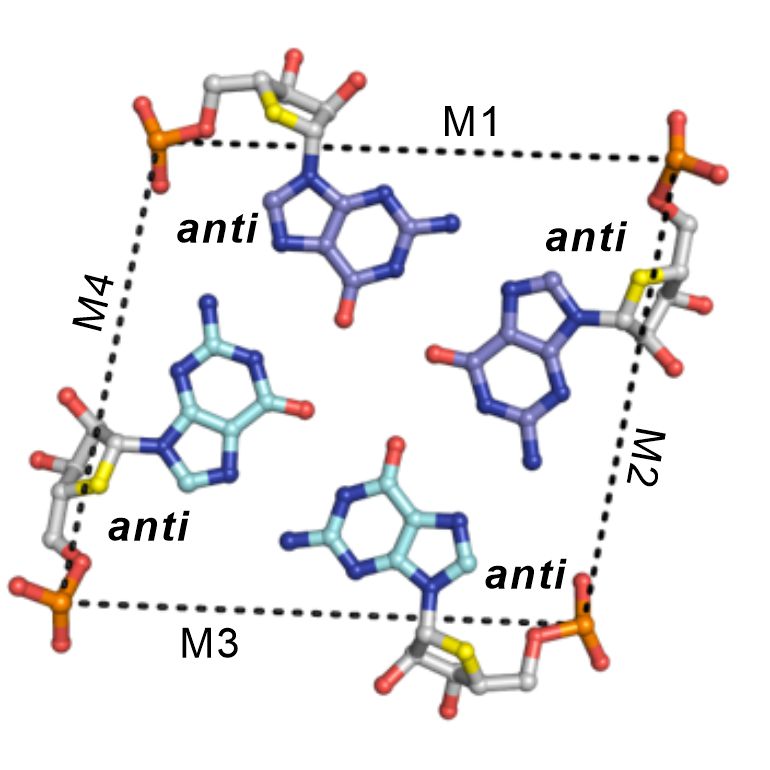

Supplement: gkae473_Supplemental_File [file gkae473_supplemental_file.docx]
